# Worms

**Published:** 1856-03

**Authors:** 


*Worms.*—Dr. Devlaux, of Brussels, has been experimenting with quinine in cases of intestinal worms, and reports (Gazette des Hop.) forty cases of the ascaris lumbricoides radically cured by this remedy in two grain doses, repeated once or twice during the day. Its indication is especially found in sickly children where the usual vermifuges would be inadmissible.

